# Activation of P2X4 receptor exacerbates acute brain injury after intracerebral hemorrhage

**DOI:** 10.1111/cns.13831

**Published:** 2022-03-30

**Authors:** Si‐Ting Wu, Jin‐Rui Han, Nan Yao, Yu‐Lin Li, Fang Zhang, Yao Shi, Fu‐Dong Shi, Zhi‐Guo Li

**Affiliations:** ^1^ Department of Neurology Tianjin Neurological Institute Tianjin Medical University General Hospital Tianjin China; ^2^ Center for Neurological Diseases China National Clinical Research Center for Neurological Diseases Beijing Tiantan Hospital Capital Medical University Beijing China

**Keywords:** hemorrhagic stroke, microglia, neuroimmunomodulation, receptors purinergic P2X4

## Abstract

**Introduction:**

Intracerebral hemorrhage (ICH) accounts for 10%–15% of all strokes and culminates in high mortality and disability. After ICH, brain injury is initiated by the mass effect of hematoma, followed by secondary cytotoxic injury from dying brain cells, hematoma disintegration, and cascading brain immune response. However, the molecular mechanism of secondary cytotoxic brain injury in ICH is not completely understood. The sensitive purinergic receptor, P2X4 receptor (P2X4R), was known to recognize extracellular free ATP released by dying cells during tissue injury.

**Aims:**

In this study, we aim to understand the role of P2X4R in acute brain injury triggered by ICH.

**Results:**

In this study, we found that the sensitive purinergic receptor, P2X4R, was upregulated in the brain of patients with ICH as well as in a mouse model of ICH induced by collagenase injection. P2X4R blockage with the specific inhibitor 5‐BDBD attenuated brain injury in ICH mice by significantly reducing brain edema, blood–brain barrier leakage, neural death, and ultimately acute neurodeficits. Further study indicated that the protective effect of P2X4R inhibition is related to decreased pro‐inflammatory activity of microglia and recruitment of peripheral immune cells into the hemorrhagic brain.

**Conclusions:**

These results suggest that the P2X4 receptor is activated by ICH stimuli which worsen brain injury following ICH.

## Graphical abstract

Intracerebral hemorrhage (ICH) triggers the upregulation of P2X4R in the brain of patients with ICH. P2X4R inhibitor 5‐BDBD can reduce brain injury such as blood–brain barrier (BBB) leakage after ICH in pro‐clinical mouse model.

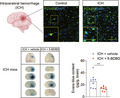

## 1 INTRODUCTION

Intracerebral hemorrhage (ICH) accounts for 10%–15% of all strokes.^1^ It is the second most common stroke type and presents a high mortality rate of 30%–50%. Moreover, 74% of the survivors remain neurologically deficient at 12 months post‐onset.^2^ In ICH, brain injury is initiated by the mass effect of hematoma^3^, ^4^, ^5^ and closely followed by secondary processes including cytotoxic damage from dying brain cells, decomposition of the hematoma releasing neurotoxic factors, and resultant immune response.^6^, ^7^, ^8^, ^9^ ICH elicits early microglia activation and is followed by mounting infiltration of peripheral immune cells into the injured brain.^10^, ^11^ However, the detailed mechanism of immune response in brain injury after ICH is only partially understood.

P2X4R, a type of the P2X receptor, is a highly kinetic and sensitive purinergic receptor that recognizes extracellular free ATP released by dying cells during tissue injury.^12^, ^13^, ^14^, ^15^ P2X4R is also a specific functional phenotype marker of microglia, thus involved in the pathogenesis of many neurological diseases.^16^, ^17^, ^18^, ^19^ P2X4R has been found to be upregulated in the microglia of multiple sclerosis patients and correspondingly in experimental autoimmune encephalomyelitis (EAE) mice where P2X4R expression was linked to the beneficial remyelination in EAE mice.^16^ While in experimental ischemic mouse models, the role of the P2X4 receptor in acute brain injury and neural repair remains controversial.^20^, ^21^ In the ICH disease paradigm, the expression and function of the P2X4 receptor have not been explored, and we examine the role of the P2X4 receptor in acute brain injury in a classic mouse model of ICH.

## 2 MATERIALS AND METHODS

### 2.1 Mice

Eight to 10‐weeks old C57BL/6 male mice were used in this study. All mice were housed in pathogen‐free conditions at an animal facility under a standardized light–dark cycle with free access to food and water. All animal experiments were approved by the Committee on the Ethics of Animal Experiments of Tianjin Neurological Institute (Tianjin, China). All animal experiments were performed in accordance with the National Institutes of Health Guide for the Care and Use of Laboratory Animals and animal data reporting followed ARRIVE guidelines 2.0 (Animal Research: Reporting of In Vivo Experiments). All surgeries were performed under anesthesia.

### 2.2 Administration of 5‐BDBD

5‐(3‐Bromophenyl)‐1,3‐dihydro‐2*H*‐benzofuro[3,2‐*e*]‐1,4‐diazepin‐2‐one (5‐BDBD, Tocris Bioscience) is a specific inhibitor of P2X4R having the ability to permeate the blood–brain barrier (BBB) into the brain.^21^, ^22^, ^23^, ^24^ In this study, the compound was dissolved in dimethyl sulfoxide at a concentration of 1 or 2 mM. Mice were orally administered 5‐BDBD (1 mg/kg body weight) or vehicle at 0 h, day 1, and day 2 post‐ICH induction. For in vitro experiments, 3 μM 5‐BDBD or the same volume of vehicle was used to treat culturing microglia isolated from WT mouse.

### 2.3 ICH induction

ICH was induced by collagenase injection in mice, as we previously published.^25^, ^26^ Mice were anesthetized with an intraperitoneal injection of 5% chloral hydrate (7 ml/kg body weight). Throughout the surgeries, a 37°C heat lamp was used to maintain the body temperature of mice. Thereafter, the mice were secured in a stereotactic frame and a needle was positioned at the coordinates 2.3 mm lateral to the midline, 0.5 mm anterior to bregma on the skull. The needle was slowly inserted to a depth of 3.5 mm beneath the skull. Mice were injected with sterile saline‐diluted 0.0375 U bacterial collagenase (Type IV‐S, Sigma) at a rate of 0.5 μl/min using an infusion pump (KD Scientific Inc). The needle was then withdrawn slowly 5 min after injection. Following the withdrawal of the needle, the burr hole in the mouse skull was sealed, and the skin incision was sutured. The recovering mice were then transferred to cages with free access to food and water.

### 2.4 Neurological deficit assessment

Two investigators blinded to mouse groupings performed modified Neurological Severity Score (mNSS) and Rota‐rod tests at day 1 and day 3 after ICH. The mNSS is 18 scale used for assessing neurological function, including motor, sensory, reflex, and balance.^27^ Each mouse was given 0–18 points score for each tested abnormality in behavior or lack of a reflex according to established scoring criteria. Finally, an overall score was given to determine the overall neurological impairment in each mouse. The Rota‐rod test evaluated the balance, grip strength, and motor coordination of mice on a rotating rod with accelerating velocity. At day 1 and day 3 post‐ICH, each mouse was tested three times per day for a maximum of 5 min in each test, with an accelerating rotational speed ranging from 0.00266 to 0.266 *g*. The time interval between trials of each mouse was 10 min, and the average latency to falling off the rotating rod was measured.

### 2.5 Brain water content assessment

At day 3 after ICH, mice were euthanized and decollated to assess brain water content. Brain tissues were divided into the left hemisphere, right hemisphere, and cerebellum apart from the head. Each of the three parts was weighed to obtain wet weight and then dried for 72 h at 65°C to get the dry weight. Finally, brain water content was calculated using the following formula: (wet weight−dry weight)/wet weight × 100%.

### 2.6 Immunofluorescence

At day 3 after ICH, mice were perfused with cold PBS followed by 4% paraformaldehyde (PFA). Brain tissues were fixed in 4% PFA overnight and then embedded in paraffin after dehydration and hyalinization. Five‐micrometer thick coronal sections were deparaffinized and rehydrated in a series of ethanol dilutions. After 30 min antigen retrieval at 95°C, brain tissue sections from ICH patients and ICH mice were permeabilized and incubated with a 5% donkey serum blocking solution and 0.3% Triton‐X 100 for 60 min. Afterward, brain sections were incubated with primary antibodies against P2X4R (Proteintech), claudin‐5 (Invitrogen), CD31 (Abcam), NeuN (Abcam), and caspase‐3 (Cell signaling technology) at 4°C overnight. After washing with PBS, slides were incubated with appropriate fluorochrome‐conjugated secondary antibodies: donkey anti‐rabbit 488 (Invitrogen), donkey anti‐mouse 488 (Invitrogen), donkey anti‐mouse 546 (Invitrogen), donkey anti‐rabbit 546 (Invitrogen), respectively, at room temperature for 60 min. Finally, all the slices were incubated with DAPI (Abcam). Images were captured with a fluorescence microscope (Model BX‐61, Olympus). The intensity of immunofluorescence and number of positive cells was quantified by Image J (U.S. National Institutes of Health).

### 2.7 Hematoma volume

The measurement of hematoma volume followed the well‐established procedures as previously described.^28^ ICH mice were euthanized at day 1 after onset, and brain sections were taken with an interval of 200 μm, starting at +2 mm to bregma and extending to −4 mm to bregma. Brain sections were stained with Modified Hematoxylin–Eosin (HE) Stain Kit (Solarbio) and captured by Cytation Cell Imaging Reader (Bio Tek). The hematoma volume was analyzed by Image J (U.S. National Institutes of Health).

### 2.8 Evans blue permeability assay

Blood–brain barrier breakdown was assessed by Evans blue permeability assay as previously described.^29^ In brief, mice were intravenously injected with 2% solution of Evans blue (Sigma‐Aldrich) in normal saline (4 ml/kg of body weight) at day 3 post‐surgery. After circulation of Evans blue for 4 h, mice were euthanized and perfused with 40 ml PBS solution. The ipsilateral brains were harvested and weighed. The brain tissue homogenates was incubated with formamide in a 60°C water bath for 72 h to extract the evans blue. After centrifugation, the Evans blue concentration was quantified photometrically by a microplate reader (Thermo Scientific) at 600 nm. The following formula was used: EB content in brain tissue (μg/g wet brain) = EB concentration (μg/ml) × formamide (ml)/wet weight (g).

### 2.9 Flow cytometry

Flow cytometry was performed to determine the expression of P2X4R and cell infiltration in the brain. Briefly, the brain was removed after perfusion with cold PBS and mechanically cut into small pieces using sharp scissors. We digested brain tissue in 1 mg/ml collagenase (Sigma) in PBS at 37°C for 30 min, and then isolated and removed the myelin in 30% percoll after 700 g for 10 min without brakes. The bottom layer was harvested and re‐suspended with 1% BSA. Single‐cell suspensions were stained with antibodies of the cell surface maker for 30 min. After washing with PBS at 800 *g* for 5 min, cell pellets were fixed and permeabilized, followed by staining with antibodies of intracellular makers. Furthermore, the primary antibody of the neuronal maker needs to combinate a secondary antibody. All antibodies were purchased from BD Bioscience, Inc. or eBioscience, Inc. unless otherwise indicated. The procedure of cell staining followed the manufacturer protocol. The following antibodies were used: CD3 (145‐ 2C11), CD45 (30‐ F11), CD11b (M1/70), F4/80 (6F12), Ly6G (1A8), CD19 (1D3), GFAP (1B4), Neural Nuclei (NeuN), P2X4R (1A5A6), IL‐6 (MP5‐20F3), IL‐10(JES5‐16E3), TNF‐α(MP6‐XT22), TGF‐β1(TW7‐16B4),donkey anti‐mouse 488 (Invitrogen).

### 2.10 Cell culture

Microglia were purified from 8 to 10 weeks naive C57BL/6 male mice. The brain tissues were minced with scissors in ice‐cold DMEM. Subsequently, the minced tissues were digested with collagenase at 37°C for 30 min. After washing and re‐suspending with 1% BSA, single cell suspensions were stained with anti‐CD45 and anti‐CD11b at 4°C for 30 min. After that, microglia (CD45^int^CD11b^+^) were isolated by a FACS Aria III flow cytometer (BD Bioscience). The purity of microglia was confirmed by flow cytometry after sorting (>99%). Isolated microglia were cultured in high glucose DMEM medium with 10% FBS and 1% penicillin/streptomycin and culture within an incubator at 5% CO_2_ and atmospheric O_2_ concentration at 37°C.

Lysed red blood cells (RBCs) were obtained as previously described.^30^ Briefly, autologous blood was washed in saline. After centrifugation and re‐suspension, RBCs were lysed by freezing the cells in liquid nitrogen for 8 min followed by denaturing at 37°C for 5 min. The freeze‐thaw was repeated three times.

At 24 h after seeding in a six‐well cell culture dish, microglia were incubated with RBCs lysate (1 µl lysate/100 µl medium) or saline for 6 h. Thereafter, microglia were treated with 5‐BDBD (3 μM) or vehicle. At day 3 after treating with 5‐BDBD or vehicle, microglia were harvested and used for flow cytometry analysis and qPCR analysis.

### 2.11 Real‐time polymerase chain reaction (PCR)

We used EZ‐press Cell to cDNA Kit PLUS (EZ Bioscience) to produce cDNA from the cell in culture. After treating with 5‐BDBD or vehicle for 3 days, microglia were collected for cell counts. Microglia of each group with the same cell counts were used to produce cDNA by EZ‐press Cell to cDNA Kit PLUS. PCR was performed on an Optical 2 Real‐Time PCR Detection System (Bio‐Rad) with the appropriate primers and SYBR Green PCR Master Mix (Roche Diagnostics). The primers used to detect gene expression are listed as follows: CCL2 (F: ATTCTGTGACCATCCCCTCAT, R: TGTATGTGCCTCTGAACCCAC), CCL5 (F: GCTGCTTTGCCTACCTCTCC, R: TCGAGTGACAAACACGACTGC), CXCL2 (F: CCAACCACCAGGCTACAGG, R: GCGTCACACTCAAGCTCTG). CXCL10 (F: CCAAGTGCTGCCGTCATTTTC, R: GGCTCGCAGGGATGATTTCAA).

### 2.12 Statistics

All values are shown as Means ± SEM. Statistical data analyses were performed using GraphPad Primes 8.0 software. The D'Agostino‐Pearson test and Shapiro–Wilk test were used to verify the normality distribution of the data. A two‐tailed unpaired Student's *t*‐test was used to determine the significance of differences between the two groups. One‐way ANOVA followed by Tukey post hoc test was used for comparisons of three or more groups. Two‐way ANOVA followed by Bonferroni post‐test was used for multiple comparisons. Significance was set at *p* < 0.05.

## 3 RESULTS

### 3.1 Upregulated P2X4R expression after ICH in humans and mice

**FIGURE 1:**
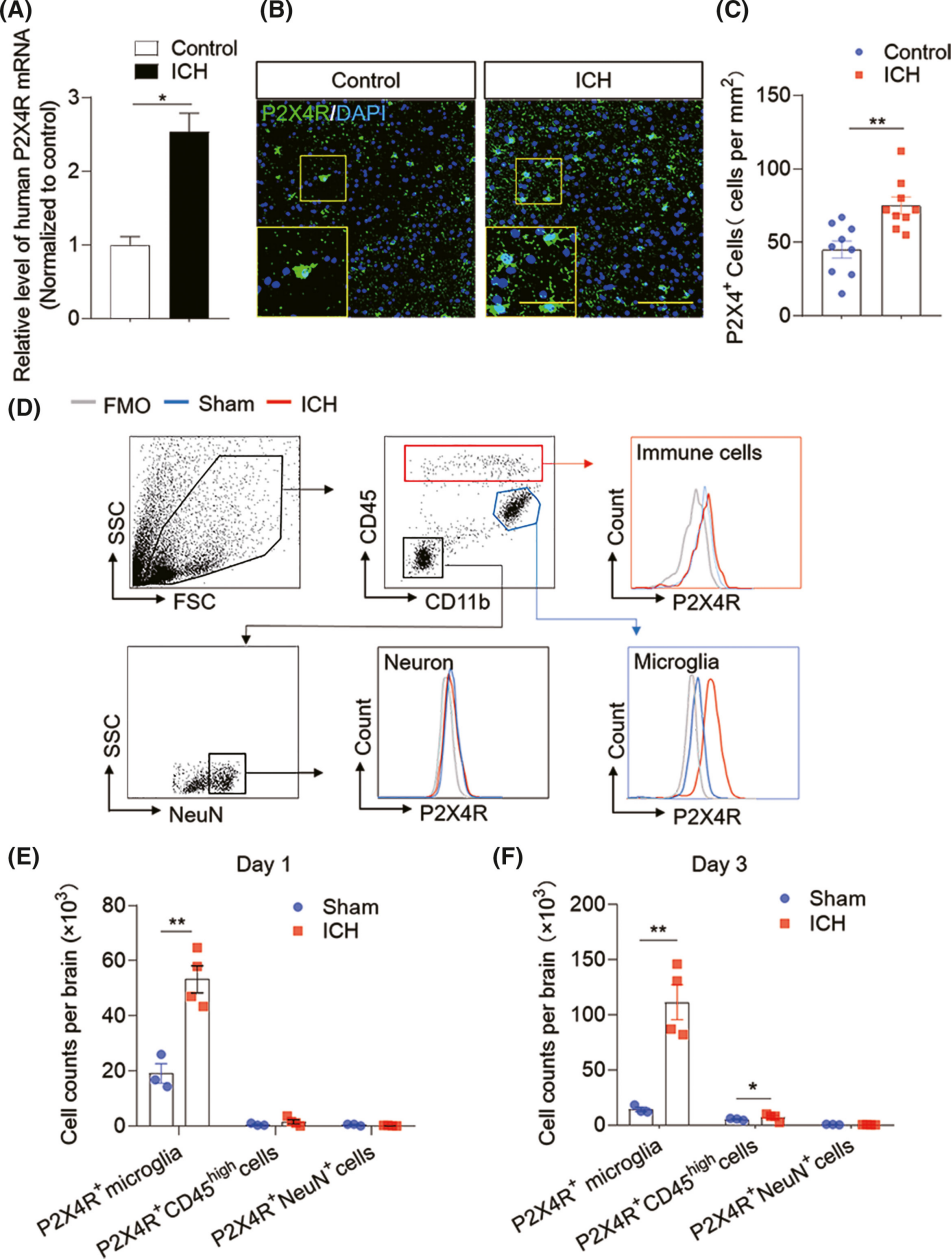
Upregulated P2X4R expression after ICH in humans and mice. C57BL/6 mice were subjected to sham or ICH surgery. Single‐cell suspensions were prepared from the ipsilateral hemisphere of mice with ICH induced by collagenase injection or sham mice at day 1 and day 3 after surgery. (A) Bar graph showing mRNA expression of human P2X4R from brain tissues proximal to the hematoma obtained from ICH patients within 24 h after ICH onset (*n* = 11) and controls (*n* = 6) (B, C) Immunostaining (B) and summarized results (C) of P2X4R in brain sections from patients ICH within 24 h after onset (*n* = 9) or controls (*n* = 9). Scale bar: 40 μm; insert: 20 μm. (D) Flow cytometry plots show the gating strategy of microglia (CD11b^+^CD45^int^), neurons (NeuN^+^), and brain‐infiltrating leukocytes (CD45^high^) cell subsets and the expression of P2X4R in these cells. (E, F) Quantification of P2X4R^+^ cells in indicated cell subsets including CD11b^+^CD45^int^ P2X4R^+^, CD11b^+^CD45^high^ P2X4R^+^, and NeuN^+^ P2X4R^+^ cells at 24 h (E) and 72 h (F) after sham operation or ICH model. *n* = 3 mice in sham group, *n* = 4 mice in ICH group. Data are presented as mean ± SEM. *p < 0.05, **p < 0.01

After ICH, brain injury is initiated by the mass effect of hematoma and perihematomal edema formation and expansion. Therefore, we collected peri‐hematomal tissues from ICH patients subjected to craniotomy surgery to remove brain hematoma within 24 h after ictus and tested for the alteration of gene expression as previously described.^31^ We found that ICH significantly increases the expression of P2X4R mRNA in injured brain tissues when comparing brain perihematomal edema tissues of ICH patients with those of healthy controls (Figure 1A). Furthermore, we observe a corresponding increased expression of P2X4R protein in the brain sections of ICH patients via immunofluorescent staining (Figure 1B,C). Together, these data depict a marked upregulation of P2X4R expression in the human brain following ICH onset.

To verify the expression pattern of P2X4R in patient samples, we used a collagenase ICH mouse model. We performed flow cytometry with the ipsilateral hemispheres of ICH mice at day 1 and 3 post‐ICH induction. As shown in Figure 1, we found that the protein levels of P2X4R in the ICH mouse brain are clearly increased compared with the sham mice at the same time points (Figure 1D–F). Analysis of gated individual P2X4R‐expressing cell subsets indicates that microglia represent the predominant P2X4R‐bearing cell subset after ICH (Figure 1E,F). These findings demonstrate that ICH triggers the upregulation of P2X4R in injured brain tissue at the acute stage of ICH, and microglia is the major cell type expressing P2X4R.

### 3.2 P2X4R exacerbates neurological deficits and brain edema after ICH in mice

**FIGURE 2:**
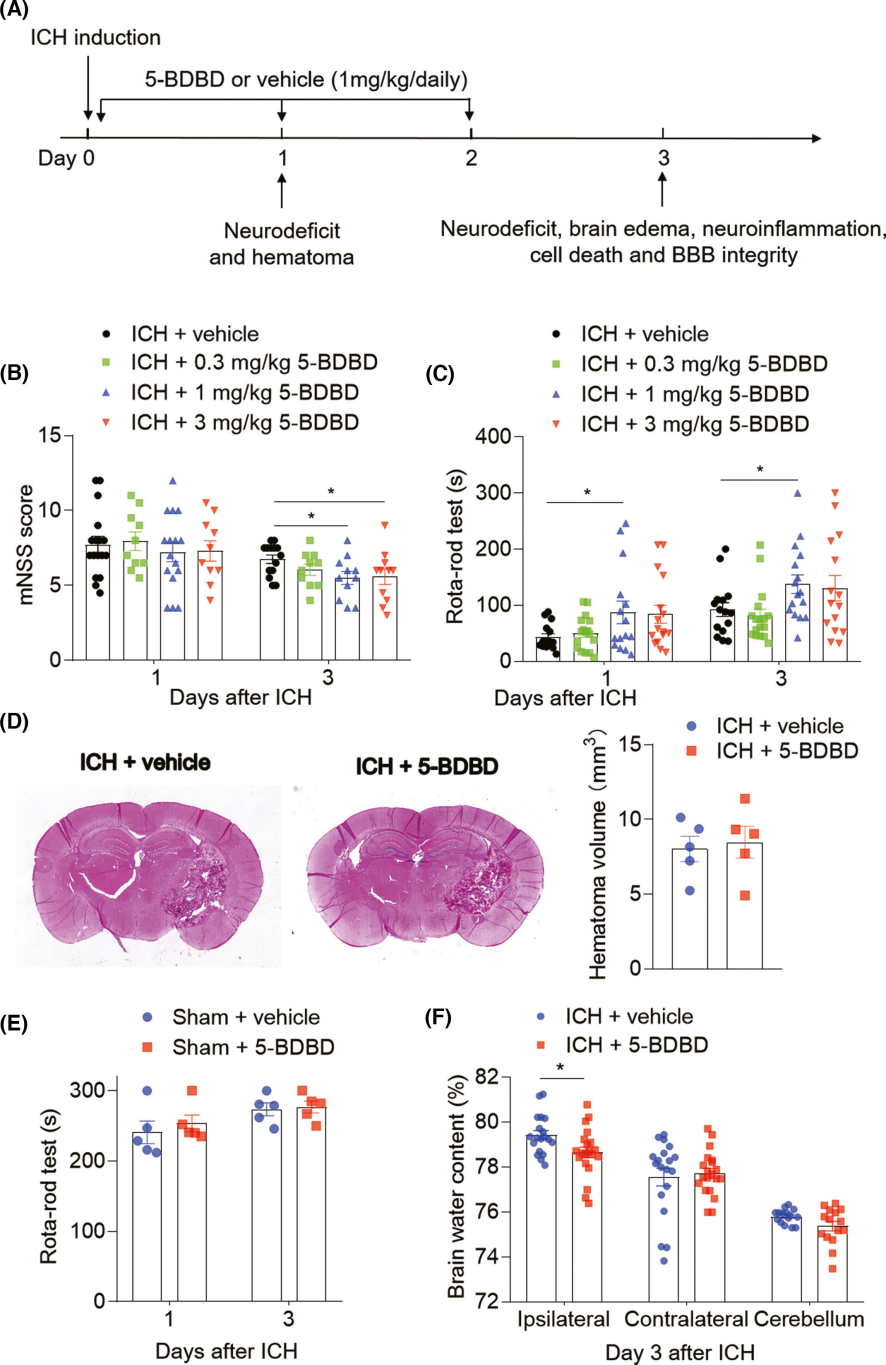
P2X4R exacerbates neurological deficits and brain edema after ICH in mice. (A) Schematic showing the regimen of P2X4R inhibitor administration and experimental design. ICH was induced in mice by injection of collagenase, immediately followed by intragastric injection of P2X4R inhibition (5‐BDBD, 1 mg/kg) or vehicle, once‐daily treatment until 2 days after surgery. Mice were subjected to neurological assessment on day 1 and day 3, brain water content quantification, magnitude of neuroinflammation, brain cell death, and BBB integrity at day 3 after ICH. (B, C) Bar graphs illustrate the indicated neurologic tests of ICH mice given vehicle, 0.3 mg/kg 5‐BDBD, 1 mg/kg 5‐BDBD and 3 mg/kg 5‐BDBD on day 1 and day 3. *n* = 10–15 mice per group. (D) HE staining images and bar graph show the hematoma volume of ICH mice treated with vehicle and 5‐BDBD. *n* = 5 mice per group. (E) Bar graphs show the Rota‐rod test of sham mice received 5‐BDBD (1 mg/kg) or vehicle. *n* = 5 mice per group. (F) At day 3 after ICH, the brain water content of the ipsilateral hemisphere, contralateral hemisphere, and cerebellum was assessed. 5‐BDBD (1 mg/kg) treatment decreased brain water content in the ipsilateral hemisphere in the ICH model. *n* = 20 mice per group. Data are presented as mean ± SEM. *p < 0.05

To determine whether P2X4R is involved in acute brain injury after ICH, we administrated ICH mice with the P2X4R inhibitor, 5‐BDBD (1 mg/kg body weight) or vehicle (DMSO) via oral gavage at 0 h, day 1 and day 2 after ICH, and examined subsequent neurodeficits and perihematomal edema in ICH mice (Figure 2A). The dose of 5‐BDBD (1 mg/kg body weight) was optimized based on the effect on neurodeficits of ICH mice (Figure 2B,C). Neurodeficits were quantified using mNSS and Rotarod testing tests at day 1 and day 3 after ICH induction. Notably, mice treated with 5‐BDBD (1 mg/kg body weight) displayed attenuated neurologic impairment at day 1 after ICH. This beneficial effect can last to day 3 after ICH (Figure 2B,C). We find that 5‐BDBD affected neither the hematoma size of ICH mice induced by collagenase injection (Figure 2D) nor the neurological function of sham control mice (Figure 2E). However, when quantifying the magnitude of brain edema by the wet/dry weight method, we found that 5‐BDBD significantly prevented the expansion of ICH‐induced brain water content in the ipsilateral hemisphere (Figure 2F). In all, these results demonstrate that P2X4R inhibition significantly reduced neurodeficits and peri‐hematomal brain edema following the onset of ICH in mice.

### 3.3 P2X4R inhibition alleviates inflammatory reaction in the brain after ICH

**FIGURE 3:**
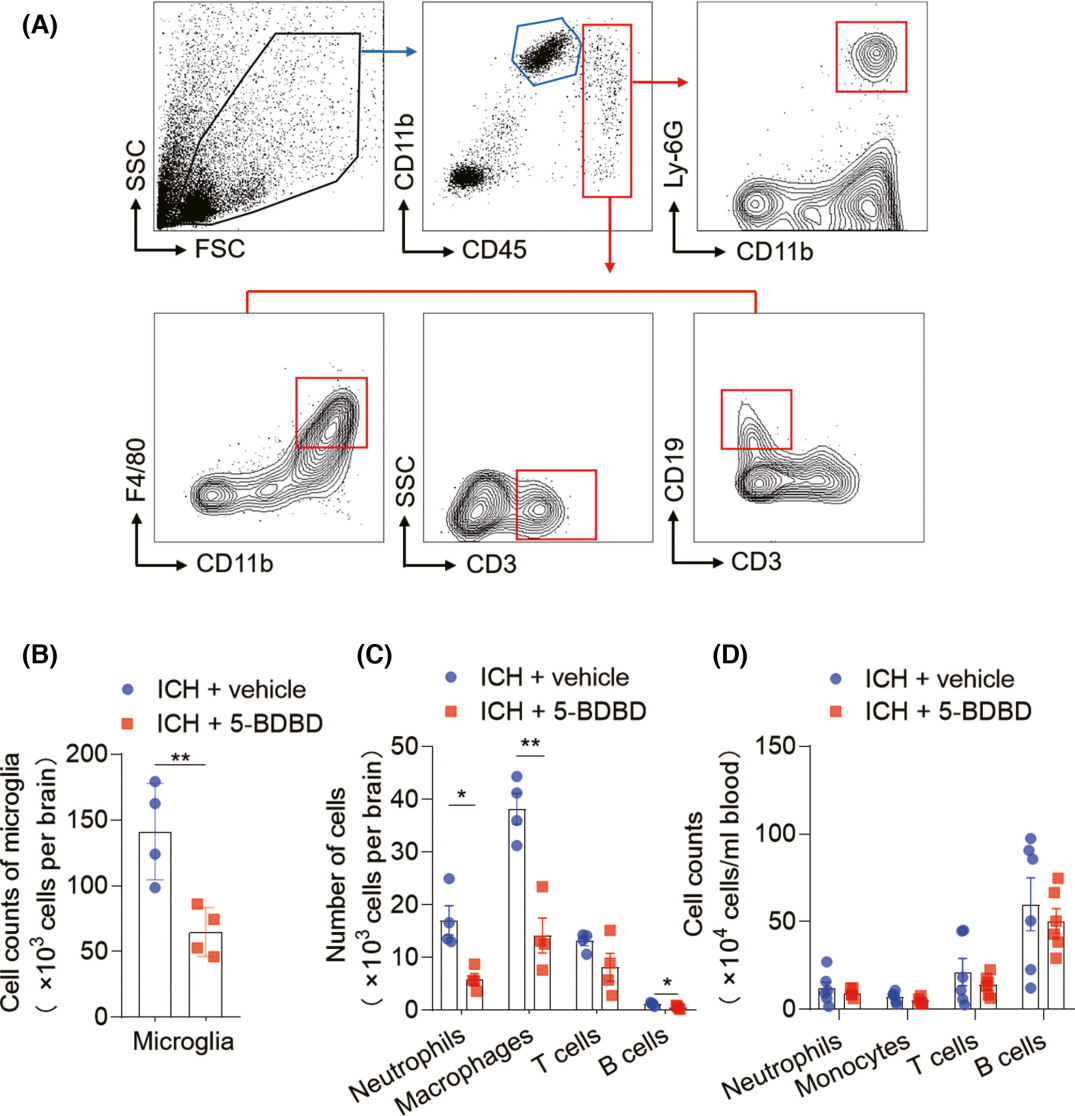
P2X4R inhibition alleviates inflammatory reaction in the brain after ICH. ICH was induced by collagenase injection and immediately followed by daily intragastric injection of P2X4R inhibition (5‐BDBD, 1 mg/kg) or vehicle until 2 days after surgery. The whole‐brain tissues were harvested to isolate single cells for flow cytometry analysis at day 3 after ICH. (A) Representative flow cytometry plots show the gating strategy of microglia (CD11b^+^CD45^int^), neutrophils (CD45^high^CD11b^+^Ly‐6G^+^), macrophages (CD45^high^CD11b^+^F4/80^+^), CD3^+^ T cells (CD45^high^CD3^+^) and B cells (CD45^high^CD3^−^CD19^+^). (B, C) Cell counts of microglia and CNS‐infiltrating neutrophils, macrophages and lymphocytes from ICH mice given vehicle or 5‐BDBD treatment at day 3 after surgery, *n* = 4 mice per group. (D) Data summaries the cell counts of leukocyte subsets in peripheral blood, *n* = 6 mice per group. Data are presented as mean ± SEM. *p < 0.05, **p < 0.01

Purinergic receptors, including P2X4R, are thought to play a vital role in activating microglia and the cascading processes of neuroinflammation and neurodegeneration in paradigms of brain injury.^32^ As such, we sought to determine whether P2X4R contributes to the brain immune response in the ICH microenvironment. By assaying the cellular components in the ICH‐afflicted brain, including brain‐infiltrating leukocytes and microglia at day 3 (Figure 3A), we found that P2X4R inhibition with 5‐BDBD dramatically reduced the counts of microglia (CD11b^+^CD45^int^), brain‐infiltrating neutrophils (CD45^high^CD11b^+^Ly‐6G^+^), macrophages (CD45^high^CD11b^+^F4/80^+^), and B cells (CD45^high^CD3^−^CD19^+^) in mice at the acute time points (Figure 3B,C). However, the leukocyte subsets in peripheral blood of ICH mice were not altered by P2X4R blockage (Figure 3D). These results suggest that P2X4R inhibition can alleviate acute brain inflammation after ICH.

### 3.4 P2X4R inhibition attenuates cell death and BBB damage after ICH

**FIGURE 4:**
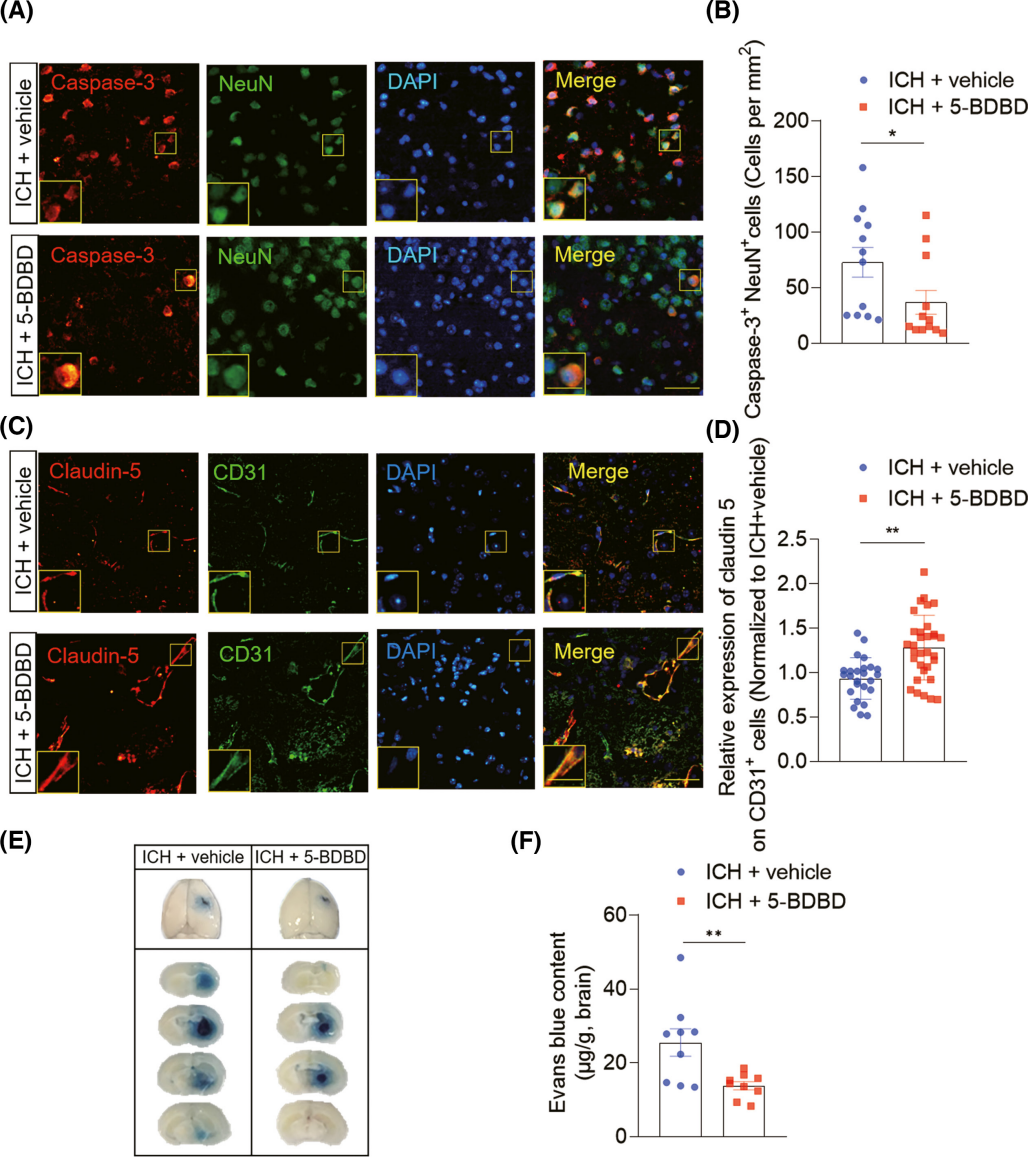
P2X4R inhibition attenuates cell death and BBB damage after ICH. Brain tissues of ICH mice receiving 5‐BDBD or vehicle treatment were collected for cell death and BBB damage analysis at day 3 in the collagenase injection surgery model. (A) Immunostaining of cleaved caspase 3(Caspase‐3; red), neuron (NeuN; green), and 4,6‐diamidino‐2‐phenylindole (DAPI; blue) were performed in brain sections from 5‐BDBD or vehicle treatment mice at 3 days post‐ICH. Scale bars: 40 μm; 20 μm (inset). (B) Quantification of the cell numbers of NeuN^+^ Caspase‐3^+^ cells in the perihematomal area of ICH mice treated with 5‐BDBD or vehicle at day 3. *n* = 12 brain slices from six mice per group. (C) Brain sections were double‐stained with tight junction strands (Claudin‐5; red) and the cluster of differentiation 31 (CD31; green) at day 3 after ICH. Scale bar: 40 μm; insert: 20 μm. (D) Summarized results show that ICH mice treated with 5‐BDBD had increased claudin‐5 integrity in immunofluorescence intensity on endothelial cells within the lesion area. *n* = 30 brain slices from eight mice per group. (E, F) Pathology staining (E) and quantification results (F) of Evans blue dye leakage on day 3 after ICH in the indicated groups. EB content in brain tissue (μg/g wet brain) = EB concentration (μg/ml) × formamide (ml)/wet weight (g). *n* = 9 per group. Data are mean ± SEM. *p < 0.05, **p < 0.01

Microglia activation and immune cell infiltration aggravate cell death and BBB injury after ICH, and the dysregulated BBB crucially contributes to edema development.^33^, ^34^, ^35^, ^36^ Thus, immunostaining analyses were performed to determine neuronal death and BBB integrity after ICH. We detected the expression of activated caspase‐3, a key mediator of cell apoptosis, in neurons from ICH mice treated with vehicle than that of 5‐BDBD, indicating attenuation of ICH‐induced neuronal death when P2XR4 is blocked (Figure 4A). Similarly, quantitation of caspase‐3^+^ NeuN^+^ cells in the peri‐hematomal area demonstrated a marked reduction of neuronal apoptosis following 5‐BDBD treatment (Figure 4B). In addition, to verify the P2X4R impact on BBB integrity after ICH, we quantified BBB permeability and expression of tight junction proteins in mice treated with P2X4R inhibition by 5‐BDBD or vehicle at day 3 after ICH. Immunofluorescent staining showed that the tight junction protein claudin‐5 on brain endothelial (CD31^+^ cells) were rescued in ICH mice treated with 5‐BDBD compared with control mice (Figure 4C,D). Similarly, 5‐BDBD‐treated ICH mice had far less extravasation of Evans Blue than those in recipients of the vehicle control (Figure 4E,F). In all, these data indicate that inhibition of P2X4R by 5‐BDBD attenuates BBB breakdown and neuron cell apoptosis after ICH.

### 3.5 Microglia contribute to the protective effect of P2X4R inhibition by 5‐BDBD

**FIGURE 5:**
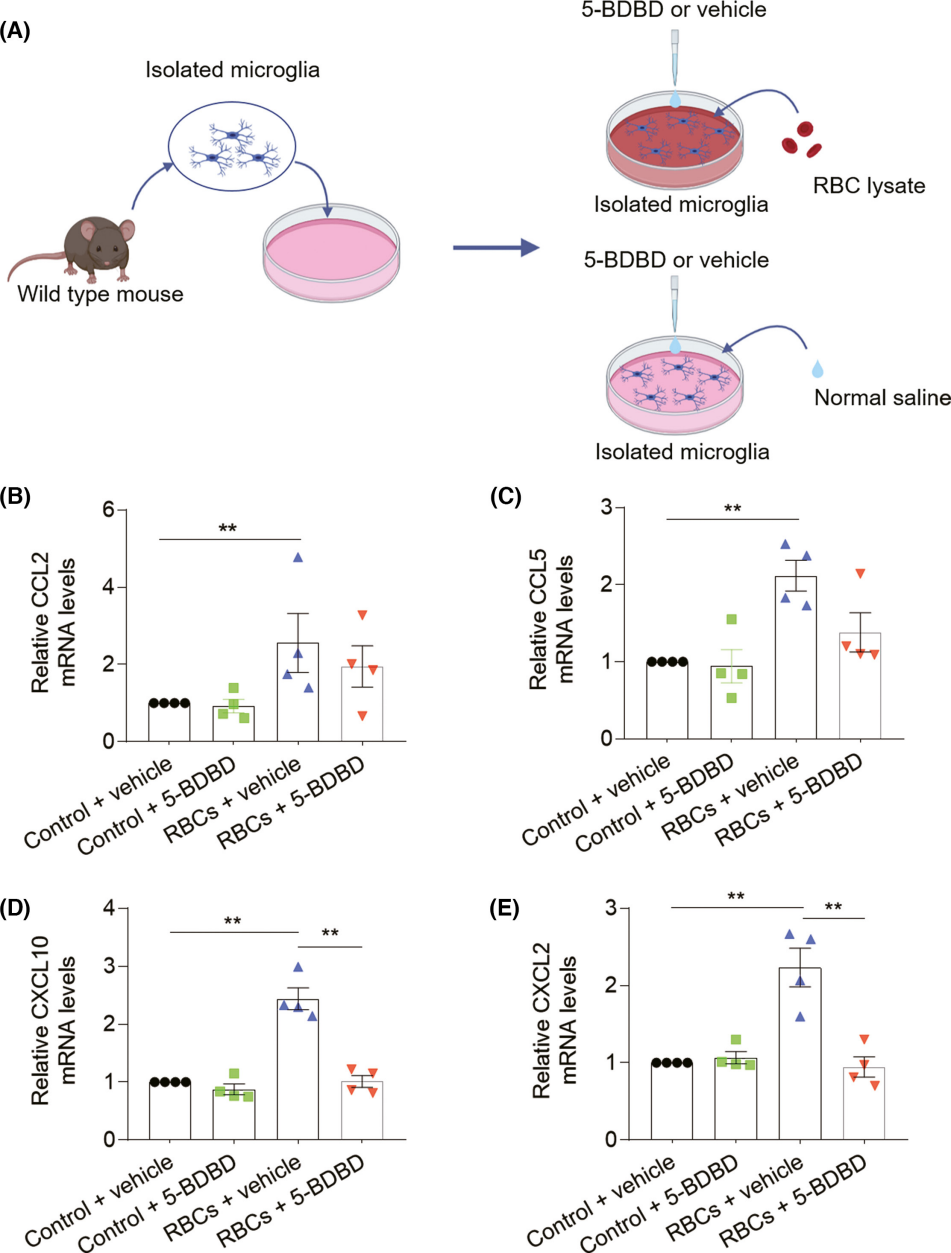
P2X4R inhibition alleviates the production of microglia‐derived chemokines after exposure to hematoma components. (A) Microglia were harvested from the brain of C57BL/6 mice and cultured in vitro for 24 h. After that, microglia were incubated with RBC lysate or normal saline for 6 h. After that, microglia were treated with P2X4R inhibition (5‐BDBD, 3 μM) or vehicle. (B–E) Representative RT‐qPCR analysis of mRNA levels showing expression of CCL2, CCL5, CXCL10, and CXCL2 in groups of microglia receiving indicated treatment at 72 h. *n* = 4 independent experiments per group. Each point data is from one independent experiment and three mice were used in every experiment. **p < 0.01. Data are presented as mean ± SEM

Since microglia are the predominant cell subset expressing P2X4R after ICH, we sought to determine what extent microglia may contribute to the protective effects conferred by 5‐BDBD. For this purpose, microglia were sorted from naive C57BL/6 male mice and cultured in vitro for 24 h, then incubated with RBCs lysate products (1 µl lysate/100 µl medium) or saline for another 6 h. Thereafter, microglia were treated with 5‐BDBD (3 μM) or vehicle for 72 h (Figure 5A). To investigate the major chemokines responsible for microglia, we assessed the expression profile of immune factors in microglia following exposure to hematoma components in vitro. PCR analysis revealed that microglial CCL2, CCL5, CXCL10, and CXCL2 were dramatically increased in cultured microglia after in vitro exposure to RBC lysate. Notably, the upregulation of CXCL10 and CXCL2 was significantly decreased after treatment with 5‐BDBD (Figure 5B–E), suggesting that the protective effect of P2X4R inhibition against ICH injury partly alleviates the production of microglia‐derived chemokines.

**FIGURE 6:**
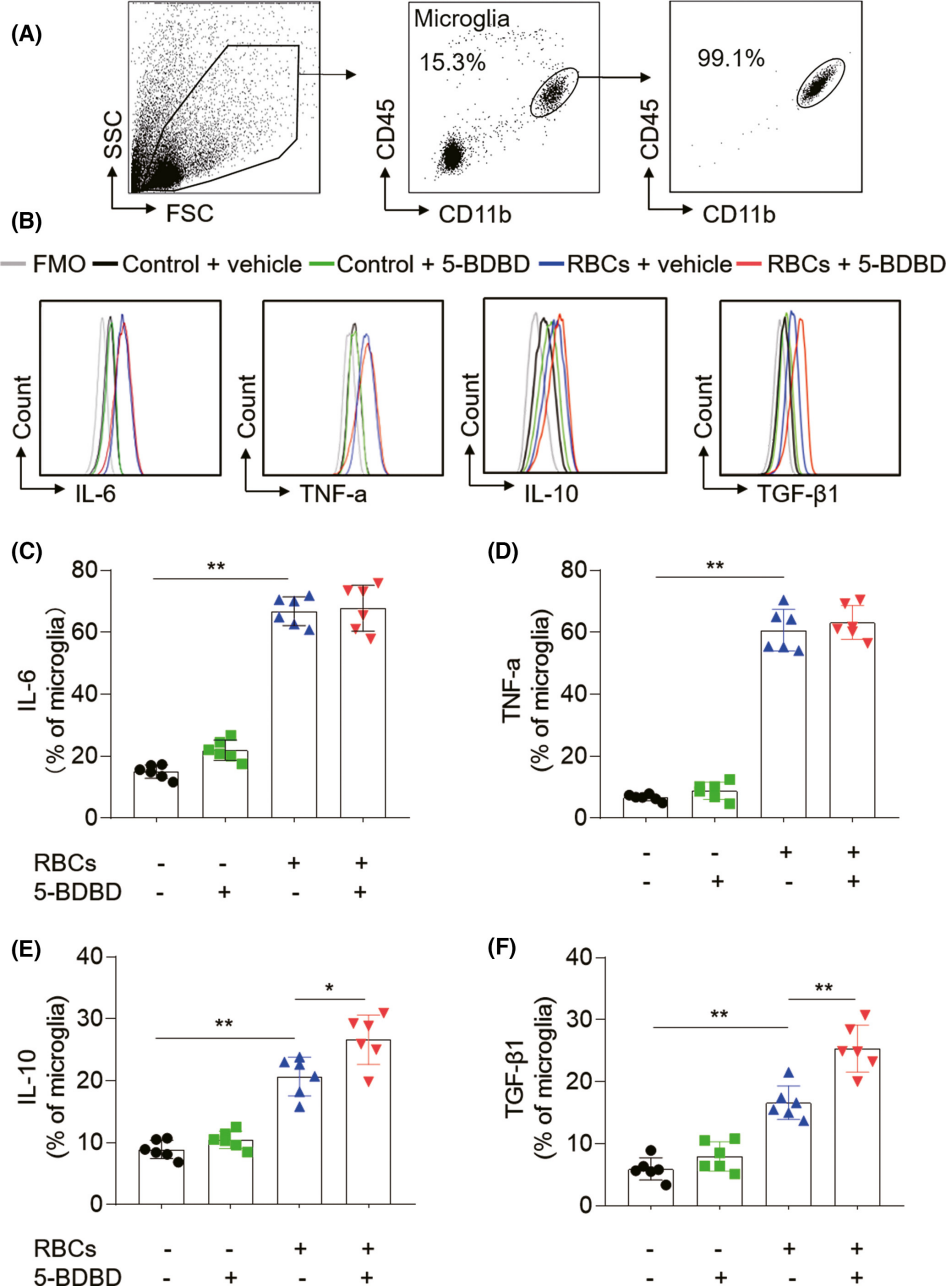
P2X4R inhibition improves the production of microglia‐derived anti‐inflammation cytokines after exposure to hematoma components. (A) Microglia were harvested from the brain of C57BL/6 mice through flow cytometry sorting. Flow cytometry plots show the strategy of microglia cluster before and after sorting. The purity of microglia was 99.1%. Then, microglia were cultured in vitro incubated with RBCs lysate. Microglia were treated with P2X4R inhibition (5‐BDBD 3 μM) or vehicle. (B–F) Flow cytometry analysis showing expression of IL‐6, TNF‐a, IL‐10, and TGF‐β1 in groups of microglia receiving indicated treatment. *n* = 6/group. Data are from three independent repeated experiments. *p < 0.05, **p < 0.01. Data are presented as mean ± SEM

Concomitantly, we also investigated the impact of 5‐BDBD on microglial cytokine releasing after in vitro RBC lysate exposure. Microglia were isolated from adult male wild‐type mice and purified by flow cytometry (Figure 6A). Our data showed that pro‐inflammatory cytokines IL‐6, TNF‐α, and anti‐inflammatory cytokines IL‐10, TGF‐β1 were upregulated in cultured microglia after RBCs lysate exposure. Interestingly, P2X4R inhibition amplified the production of microglia‐derived anti‐inflammation cytokines IL‐10 and TGF‐β1 (Figure 6B–F). Together, these findings suggest that microglia contribute to the protective effect of P2X4R inhibition against ICH injury.

## 4 DISCUSSION

Besides primary neural death, ICH also elicits secondary brain injuries, including brain edema, BBB leakage, and local brain or systemic immune response, all contributing toward its notable morbidity and mortality. However, the molecular mechanisms linking primary neural death and secondary brain injuries are not well understood. In this study, we found that ICH induced brain expression of P2X4R in both humans and mice. P2X4R can sense extracellular free ATP passively released by dying cells after tissue injury.^37^, ^38^ By inhibiting P2X4R with 5‐BDBD, our study indicates that 5‐BDBD attenuates neuroinflammation, BBB leakage, neural death, and neurodeficits in ICH mice. These study results provide new evidence pointing to P2X4R‐mediated exacerbation of acute brain injury after ICH.

The P2X4 receptor regulates the immune response of microglia and is related to neural injury in different contexts of brain disease.^39^ In ischemic stroke, P2X4R genetic knockout or P2X4R blockage reduced the infiltration of leukocytes into the brain and attenuated BBB damage, ultimately improving neurodeficits.^20^, ^21^ However, P2X4R inhibition exacerbated the neurological outcome in the mouse model of multiple sclerosis. Further studies demonstrate that P2X4R blockage promoted pro‐inflammatory activation of microglia and reduced their secretion of BDNF, which inhibits myelin phagocytosis and oligodendrocyte differentiation.^16^, ^37^, ^40^

In this study, we also find that microglia are the major P2X4R expressing cells in the brain after ICH. However, our study indicates that P2X4R inhibits the secretion of anti‐inflammatory cytokines of microglia following hemorrhage, which may enhance inflammatory brain injury. Notably, other cells may also contribute to the effect of 5‐BDBD in ICH mice, as we find that less portion of brain infiltrating immune cells also express P2X4R in the injured ICH brain at day 3 after ICH. Further studies are needed to uncover the effects of 5‐BDBD on other P2X4R^+^ cells and long‐term outcomes in ICH. In addition, only male mice were used in this study. As there are gender differences on stroke outcome, it is unclear whether and to what extent that 5‐BDBD affects ICH outcome in female mice. For example, estrogen is known to have neuroprotective effects after stroke as estrogen promotes the activation of microglial endoplasmic reticulum with an anti‐inflammatory properties in female.^41^ It is also reported that female mice have smaller infarct volume than male mice in ischemic stroke.^42^

In conclusion, P2X4R in the brain is upregulated and is a potential target to intervene the acute brain injury after ICH.

## CONFLICT OF INTEREST

The authors declare no conflict of interest.

## Data Availability

The data that support the findings of this study are available from the corresponding author upon reasonable request.
